# Eukaryotic Protein Recruitment into the *Chlamydia* Inclusion: Implications for Survival and Growth

**DOI:** 10.1371/journal.pone.0036843

**Published:** 2012-05-09

**Authors:** Eric Soupene, James Rothschild, Frans A. Kuypers, Deborah Dean

**Affiliations:** 1 Center for Sickle Cell Disease and Thalassemia, Children's Hospital Oakland Research Institute, Oakland, California, United States of America; 2 Center for Immunobiology and Vaccine Development, Children's Hospital Oakland Research Institute, Oakland, California, United States of America; 3 Graduate Program in Bioengineering, University of California, Berkeley, California, United States of America; 4 Graduate Program in Bioengineering, University of California San Francisco, San Francisco, California, United States of America; 5 Department of Medicine, University of California San Francisco, San Francisco, California, United States of America; University of Louisville, United States of America

## Abstract

*Chlamydia trachomatis* (*Ct*) is an obligate intracellular human pathogen that multiplies within a parasitophorous vacuole called an inclusion. We report that the location of several host-cell proteins present in the cytosol, the nucleus, and membranes was altered during *Ct* development. The acyl-CoA synthetase enzyme ACSL3 and the soluble acyl-CoA binding protein ACBD6 were mobilized from organelle membranes and the nucleus, respectively, into the lumen of the inclusion. The nuclear protein ZNF23, a pro-apoptosis factor, was also translocated into the inclusion lumen. ZNF23, among other proteins, might be targeted by *Ct* to inhibit host cell apoptosis, thereby enabling bacterial survival. In contrast, the acyl-CoA:lysophosphatidylcholine acyltransferase LPCAT1, an endoplasmic reticulum membrane protein, was recruited to the inclusion membrane. The coordinated action of ACBD6, ACSL3 and LPCAT1 likely supports remodeling and scavenging of host lipids into bacterial-specific moieties essential to *Ct* growth. To our knowledge, these are the first identified host proteins known to be intercepted and translocated into the inclusion.

## Introduction

*Chlamydia trachomatis* (*Ct*) is a bacterium that is responsible for the majority of sexually transmitted diseases and preventable ocular blindness in the world today [1]. Upon entry into the host cell, *Ct* undergoes rapid transformation from a metabolically dormant infectious particle, the elementary body (EB), to a replicative non-infectious form called the reticulate body (RB). Early during intra-cellular development, the RBs multiply by binary fission protected inside a newly formed parasitophorous vacuole called an inclusion [2]–[5]. This obligate intracellular organism has a small genome (1.05 Mb) with about a thousand predicted ORFs [6]–[8].

Confined in the inclusion, the bacteria intercept nutrients and metabolites of the host cell. Several bacterial produced systems, such as ABC transporters and a type III secretion system, are essential to the survival of the pathogen [5], [9]–[11]. In particular, host-derived lipids, including ceramide and sphingomyelin, are scavenged to sustain the expansion of the membrane surrounding the inclusion and the membranes of the multiplying bacteria [5], [12]–[21]. The RBs lack *de novo* synthesis for some glycerophospholipids, such as phosphatidylcholine (PC). Although PC is exclusively synthesized in the endoplasmic reticulum (ER) membrane of the host cells, it is the second most abundant lipid present in both EB and RB membranes and accounts for 40% of total bacterial lipids [22].

The PC molecular species composition of the inclusion membrane is similar to the membrane of the host cells but not of the EB and RB membranes, which contain PC species unique to the organism. Bacterial PC is characterized by the presence of branched-fatty acids at the sn-2 position that do not exist in eukaryotic lipids and host membranes [22]. Thus, host PC molecules are intercepted and modified to new species before their incorporation into the bacterial membranes. These findings indicate that a de-acylation/re-acylation cycle of host PC molecules into bacterial PC species is occurring, and is probably taking place at the membrane of the inclusion (reviewed in [5]). This remodeling cycle, also know as the Lands' pathway, is essential for maintaining the composition of eukaryotic membranes [23]. For an intracellular organism requiring host lipids for its survival, the host Lands' pathway enzymes provide an alternate mechanism to generate specific bacterial lipid species.

We report that long-chain acyl-CoA synthetase, ACSL3, a membrane-bound protein of the mitochondria and Golgi apparatus [24], was recruited into the lumen of the *Ct* inclusions in human cells. ACSL3 activates long-chain fatty acids, present at the sn-2 position of lipids, by catalyzing the ATP-dependent esterification to acyl-CoAs [25], In addition, an acyl-CoA binding protein, ACBD6, with high specificity for long-chain acyl-CoAs species abundant at the sn-2 [26], was also detected inside the inclusion. ACBD6 rapidly disappeared from the nucleus of infected cells as did the nuclear zinc finger protein, ZNF23, and both were localized solely in the lumen of the inclusion as *Ct* development progressed. ZNF23 is a repressor of a cell division and a pro-apoptotic factor [27], [28], and can interact with ACBD6. The ER-bound acyl-CoA:lysophosphatidylcholine acyltransferase 1 (LPCAT1) enzyme was mobilized to the inclusion membrane but not to the lumen. LPCAT1 catalyses the second step of the Lands' pathway and transfers long-chain acyl-CoAs to the sn-2 position of lysoPC to form PC [29], [30].

We propose that sequestration of ZNF23 into the *Ct* inclusion is another example of the bacterial-induced blockade mechanism of host cell-death [20], [31]–[36]. Our results establish that, in addition to host-derived lipids and lipid bodies, lipid metabolic enzymes of the host cell are also recruited into the inclusion. Furthermore, lipids droplets that have been modified with membrane-bound bacterial proteins have been identified in the inclusion lumen [18] and could represent a vehicle to traffic ACSL3 and ACBD6 into the lumen of the *Ct* vacuole. Mobilization of LPCAT1, also identified in lipid droplets [37], to the inclusion membrane might provide *Ct* with the ability to remodel PC into the lipid species it requires.

## Results

### Long chain Acyl-CoA synthetase 3 (ACSL3) is recruited into the *Ct* inclusion lumen

**Figure 1:**
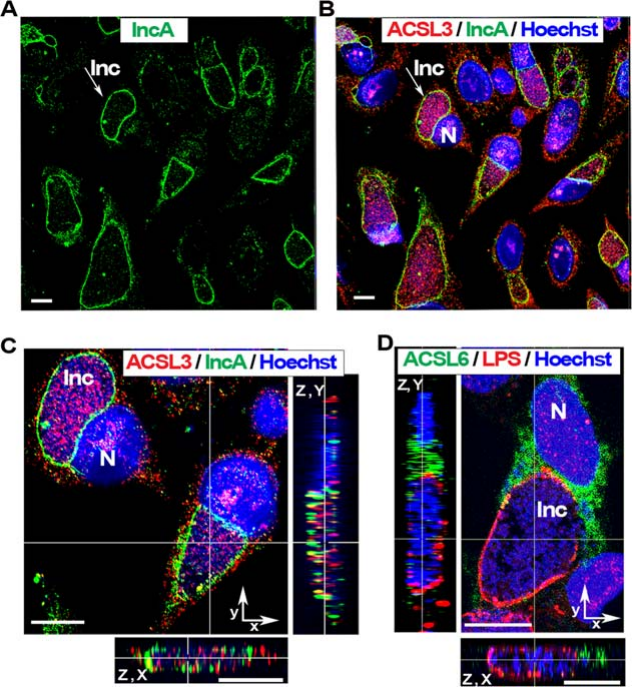
ACSL3 but not ACSL6 is recruited into the *Ct* inclusion. HeLa cells were grown on coverslips and infected with *Ct* strain D at a MOI of 1 in cell culture medium without antibiotics and cycloheximide. Cells were fixed 36 hr post- infection. DNA was detected with Hoechst 33258 dye (blue). Bacteria were detected with a rabbit antibody against IncA, which was stained with an anti-rabbit antibody labeled with AlexaFluor®488 revealing the inclusion membrane (green, panels A to C) or with a mouse antibody against LPS, which was stained with Cy^TM^3-conjugated anti-mouse antibody (red, panel D). Long-chain acyl-CoA synthetase 3, ACSL3, was detected with a mouse monoclonal antibody (red, panel B and C) and ACSL6 protein was detected with a rabbit antibody (green, panel D). The inclusion membrane of one representative inclusion is indicated with an arrow in panel A and B. Panel C displays a cropped portion of the image shown in panel B. Insets in panel C and D display the orthogonal (z, x) and (z, y) views of the deconvolved Z stack merged images. Images were taken with a Zeiss LSM710 confocal microscope at 63x magnification. The bars in panels and insets represent 10 µm. Note that the two insets of panel C were slightly scaled up compare to the main image. Nuclei and inclusion are indicated with N and Inc, respectively. ACSL3 protein was detected inside the inclusion (panel B and C) and ACSL6 was not (panel D).

Among the different members of the ACSL family, member 3 and 6 have a strong preference for activation of long-chain fatty acids toward synthesis of the glycerophospholipid phosphatidylcholine [38], [39]. ACLS3 and ACSL6 are membrane proteins associated with different organelles, such as the ER and Golgi apparatus [24], [25]. In HeLa cells infected with *Ct* strain D for 36 hr, ACLS3 protein was detected in the inclusion lumen (Figure 1, panel A to C). The membrane surrounding the inclusion was identified by staining for the bacterial protein IncA [11], [40]–[42] (Figure 1, panel A); the ACSL3 protein was detected with a monoclonal antibody, which did not cross-react with *Ct* proteins (Figure S1 and S2). However, the ACSL6 protein was not detected in the inclusion lumen (Figure 1, panel D). Bacteria were detected with an anti-LPS antibody. ACSL6 was produced in infected HeLa cells but it did not localize in the *Ct* inclusion lumen. In cells expressing a GFP-ACSL6 recombinant protein and infected with *Ct* strain L_2_, the GFP signal was also not detected in the inclusion (Figure S3). As shown in panel C of Figure 1, analysis of the deconvolved Z stack merged image established that the ACSL3 protein was not associated with the membrane of the inclusion and did not co-localize with the bacterium but was adjacent to it in the lumen of the inclusion. Even at late stages of development and in cells occupied by very large inclusions, only ACSL3 was located inside the inclusion, while both ACLS3 and ACLS6 were present in the cytosol.

### Acyl-CoA:lysoPC acyltransferase 1 (LPCAT1) is mobilized to the *Ct* inclusion membrane

**Figure 2:**
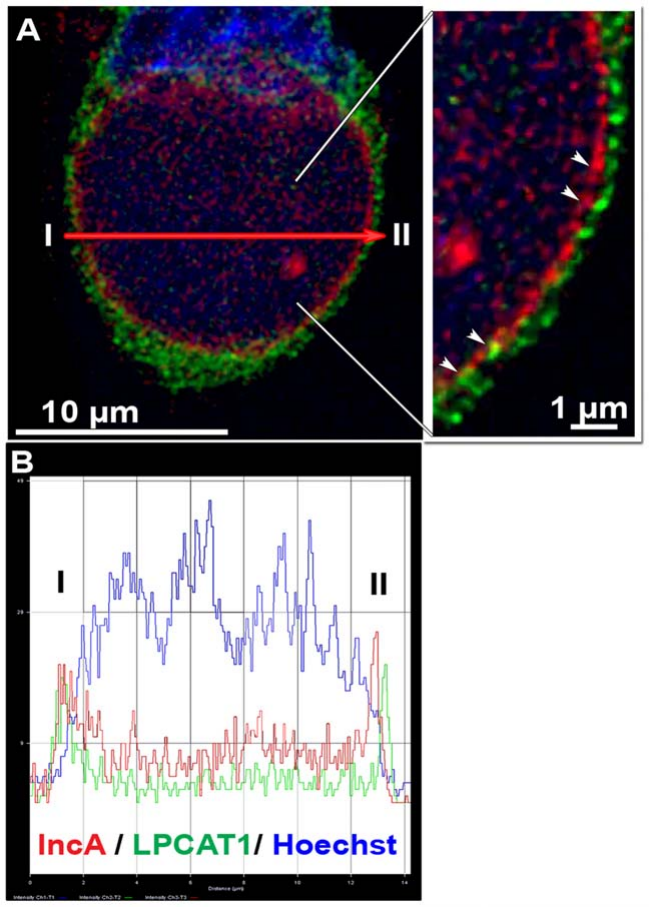
LPCAT1 enzyme is tightly associated with the inclusion membrane. HeLa cells were grown on coverslips in cell culture medium without antibiotics and cycloheximide, and were transfected with a DNA construct expressing a GFP protein fused to acyl-CoA:lysophosphatidylcholine acyltransferase 1, LPCAT1. After 24 hr, cells were infected with *Ct* strain D at a MOI of 1 and were fixed 44 hr post-infection. DNA was detected with the Hoechst dye (blue) and the bacteria were detected with a rabbit antibody against IncA revealing the inclusion membrane (red). A deconvolved Z stack merged image with the GFP signal is shown in panel A. A cropped section of the image is displayed in the inset. White arrowheads indicate the position of the inclusion membrane. The tight association of LPCAT1with the inclusion membrane and overlap of the IncA and GFP signals, detected as yellow dots, can be observed in the inset. Panel B shows traces of the intensity of the signal for DNA, IncA and LPCAT1 (y axis) plotted in function of the distance (x axis) from area I to II as indicated by a red arrow on panel A. GFP and IncA signals co-localized in area I but not in area II. Images were taken with a Zeiss LSM710 confocal microscope at 63x magnification. The bars in the panel and the inset represent 10 and 1 µm, respectively.

Several acyl-Co:lysoPhosphatidyl acyltransferases (LPLATs) have been identified in mammals but few enzymes are specific for the acceptor species lysophosphatidylcholine (LPC) [43], [44]. LPCAT1 is the re-acylating enzyme for PC in the ER and plasma membranes [29], [30], and also supports PC formation in the lipid mono-layer, which surrounds the lipid droplet [37]. These lipid bodies are formed from the outer lipid bi-layer of the ER [45]. In HeLa cells expressing a GFP-LPCAT1 fusion protein and infected with *Ct* strain D for 44 hr, LPCAT1 was not detected in the inclusion but it was tightly associated to the membrane of the inclusion (Figure 2, panel A and inset). Although the GFP signal appeared to co-localize with the bacterial IncA protein in the membrane surrounding the inclusion, the two signals were distinct (Figure 2, inset and panel B). The pattern observed with the GFP-recombinant protein in infected cells indicated that LPCAT1 is located in, or at the proximity of the lipid-rich network surrounding the inclusion [14], [18]. This reticulate structure, distinct from the ER and from the inclusion membrane, represents a source of lipid-rich vesicles, such as lipid droplets, that are engulfed in the inclusion.

### Acyl-CoA binding protein 6 (ACBD6) is a nuclear protein recruited into the *Ct* inclusion lumen

**Figure 3:**
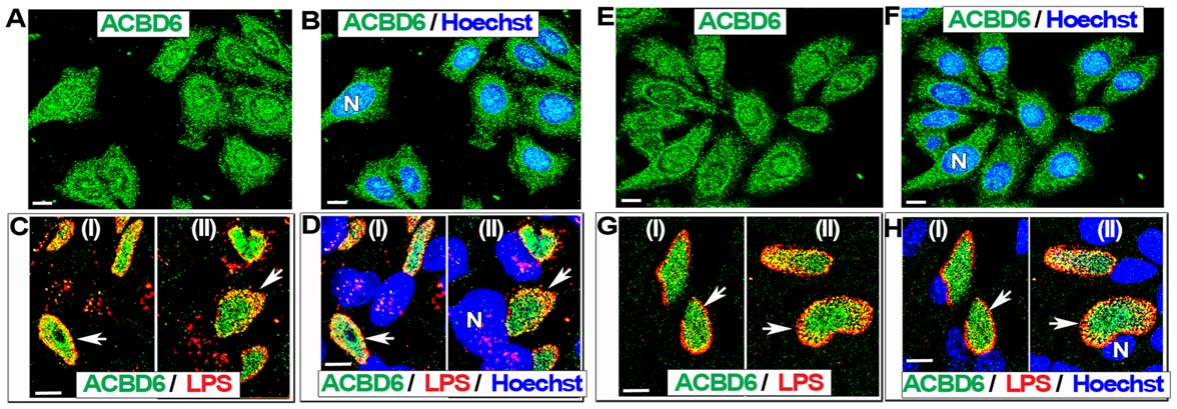
ACBD6 is mobilized into the *Ct*-inclusion lumen. Cells were grown on coverslips in cell culture medium without antibiotics and cycloheximide. Near confluent cells were fixed in methanol (panel A, B, E and F) or were infected with *Ct* strain D at a MOI of 1 and fixed 36 hr post-infection (panel C, D, G and H). Results for HeLa cells are shown in panel A to D and those obtained with HEp-2 cells are shown in panel E to H. Human ABCD6 protein was detected with an affinity-purified polyclonal antibody (green). In infected cells, bacterial LPS was detected with a mouse monoclonal antibody (red), and merged images with ACBD6 detection are shown in panel C and G. DNA was stained with the Hoechst dye (blue). In un-infected cells, merged images with stained DNA are shown in panel B and F. In infected cells, merged images with stained DNA of the host and of *Ct* cells are shown in panel D and H. In un-infected cells, ACBD6 was detected in the nucleus and the cytosol (panel A and E). In infected cells, ACBD6 protein was detected inside *Ct* inclusions (panel C and G) and not in the nuclei (panel D and H). Images were taken with a Zeiss LSM710 confocal microscope at 63x magnification. The bars in panels represent 10 µm. Representative inclusions are indicated with white arrows and nuclei by a capital N.

**Figure 4:**
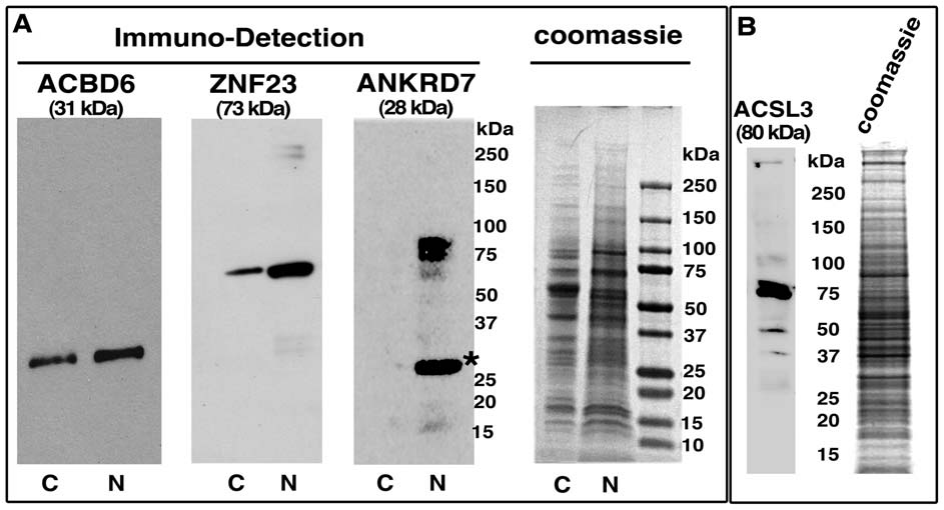
Western immunological analysis of uninfected cells. HeLa cells were grown to near confluence in the same medium that was used to perform the infection with *Ct* but cells were not infected. Panel A. Nuclear and cytosolic samples were obtained as described in the Method section. As confirmed by coomassie blue staining (right panel), an equal amount of proteins (10 µg) of the nuclear (lane N) and cytosolic (lane C) samples were loaded in each lane of a SDS-polyacrylamide 4–16 % gradient gels. After migration, the proteins were transferred on PVDF membranes that were blotted with antibody against ACBD6, ZNF23 or ANKRD7. ANKRD7 is a known nuclear protein and was used as a control [66]. The molecular mass standard (Precision Plus Protein Dual-Stained, Bio-Rad) is indicated on the right of the panels. A band of the predicted molecular mass of 32 kDa and 64 kDa was detected for ABCD6 and ZNF23 respectively, in the cytosolic and nuclear samples. A major band of the expected size of 30 kDa (asterisk) was detected for ANKRD7 in the nuclear extract. Panel B. ACSL3 protein was detected with the mouse anti-ACSL3 antibody in a total protein extract of HeLa cells as described in panel A.

**Figure 5:**
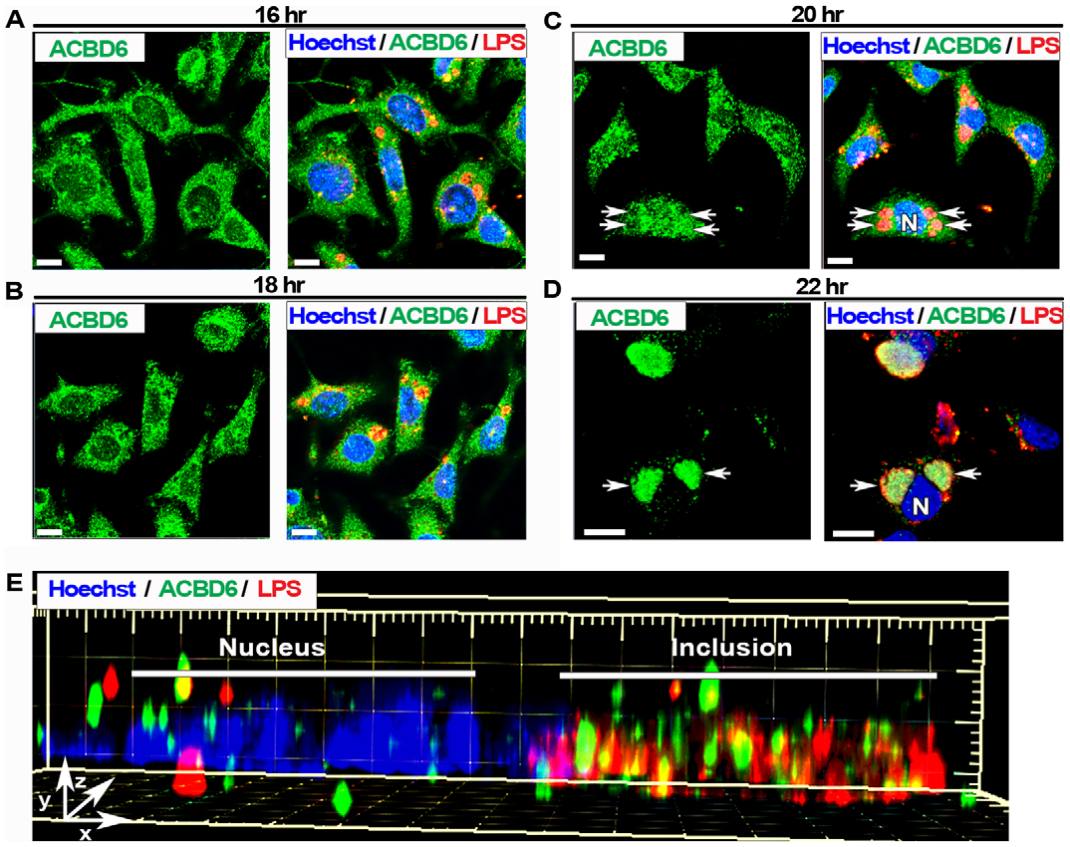
Redistribution of ACBD6 from the nucleus and cytosol into the inclusion lumen during *Ct* development. HeLa cells were grown on coverslips and infected with *Ct* strain D at a MOI of 1 in cell culture medium without antibiotics and cycloheximide. After infection, cells were fixed at the indicated time (panel A to D). ACBD6 (green), *Ct* LPS (red) and DNA (Hoechst dye, blue) were detected as described in legend of Figure 3. For each time point, the staining obtained for ACDB6 and the merge image obtained for ACBD6, LPS and DNA are shown on the left and right on each panel, respectively. Deconvolved Z stack images were analyzed with the Imaris Software package. Panel E shows the longitudinal Surpass view (x, y, z) of the distribution of ACBD6, LPS and DNA in one infected-cell 24 hr post-infection. Note that ACBD6 was detected inside the same space occupied by the bacteria in the inclusion, but did not co-localize with the LPS signal. Images were taken with a Zeiss LSM710 confocal microscope at 63x magnification. The bars in panels represent 10 µm. Representative inclusions are indicated with white arrows and nuclei by a capital N.

**Figure 6:**
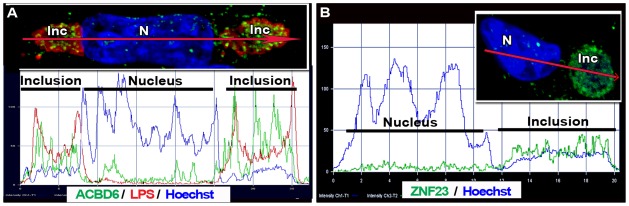
Intensity distribution profiles of ACBD6 and ZNF23 in *Ct* infected cells. HeLa cells were grown on coverslips and infected in cell culture medium without antibiotics and cycloheximide. Results obtained for ACBD6 in cells infected with *Ct* strain D 24 hr post-infection, and for ZNF23 in cells infected with strain E 18 hr post-infection, are shown in panel A and B, respectively. The images were analyzed with ZEN 2010 software (Zeiss LSM710). Traces of the intensity of the signal (y axis) were plotted in function of the distance (x axis) of the section of the cell indicated by a red arrow (from left to right). Panel A shows the merged image of ACBD6 (green), LPS (red) and DNA (blue) in a cell with two inclusions. Panel B displays the merged image of the signal for ZNF23 (green) and DNA (blue). Note that the *Ct* inclusion (Inc) can be detected with the Hoechst dye, which also stained bacterial DNA.

ACBD6 is a soluble acyl-CoA binding protein with preference for long-chain un-saturated species abundant at the sn-2 position of PC [26]. ABCD6 is highly expressed in the cytosol of progenitor stem cells but it is also present in the nucleus [26]. We also detected ABCD6 in the nucleus of HeLa cells (Figure 3, panel A and B; Figure 4, panel A) and HEp-2 cells (Figure 3, panel E and F). During infection by *Ct*, the localization of ABCD6 was dramatically altered and after 22 hr, no signal for the protein was detected outside of the *Ct* inclusion. In HeLa cells infected with *Ct* strain D, the protein signal disappeared from the cytosol and nuclei, and was detected in the inclusion lumen (Figure 3, panel C and D; Figure 5; Figure 6, panel A). This result was confirmed in HEp-2 cells (Figure 3, panel G and H). The specificity of the immunological detection of ACBD6 by the affinity-purified antibody we used, was established by the detection of a single band in protein extracts of HeLa cells (Figure 4, panel A), the competition of the detection on fixed cells by an ACBD6 synthetic peptide (Figure S4), and by the lack of cross-reactivity of the antibody with total protein extracts of *Ct* strains D and L_2_ (Figure S1 and S2). Furthermore, analysis of deconvolved Z stack merged images established that ABCD6 had been recruited into the inclusion lumen since it did not co-localize with the bacterial LPS signal (Figure 5, panel E).

Given the crucial role of Golgi-derived lipids in the expansion of the inclusion, we investigated member 3 of the ACBD family. Like ACBD6, ABCD3 is a soluble protein which binds acyl-CoAs, but it is often associated with the Golgi apparatus by interaction with the Golgi-membrane protein giantin [46]. In uninfected cells, ACBD3 was detected at the periphery of the nuclei (Figure S5, panel A). In infected cells, the signal intensity appeared weaker and, in cells with large inclusions, which occupied most of the cytosol, the protein was pushed aside (Figure S5, panel B). Thus, fragmentation of the Golgi in the infected cells might affect ACBD3 distribution [47], [48], but it was not recruited into the inclusion.

### The Nuclear Pro-Apoptotic ZNF23 protein is recruited into the *Ct* inclusion lumen

**Figure 7:**
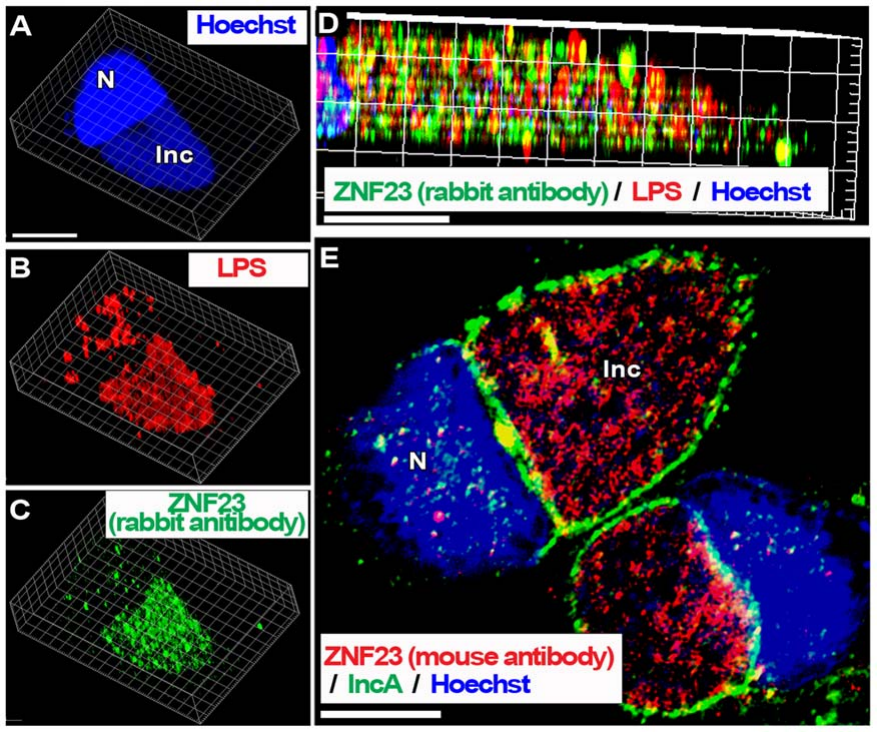
The nuclear ZNF23 protein is mobilized into the lumen of the *Ct* inclusion. HeLa cells were grown on coverslips and infected in cell culture medium without antibiotics and cycloheximide. Cells were infected at a MOI of 1 with *Ct* strain L_2_ (panel A to D) and with D (panel E) and were fixed 36 hr post-infection. DNA was detected with the Hoechst dye (blue). In cells infected with L2, the bacteria were detected with a mouse antibody against LPS (red, panel B and D) and the ZNF23 protein was detected with a rabbit antibody (green, panel C and D). Z stack images were deconvolved and single channel Surpass 3D view of a representative infected cell is shown in panel A to C. Cropped longitudinal view of the Surpass (x, y, z) view of the merged image is shown in panel D. In Panel E, the inclusion membrane was detected with a rabbit antibody against the bacterial IncA protein (green) and the ZNF23 protein was detected with a mouse antibody (red), and the merged image with Hoechst staining is shown. Images were taken with a Zeiss LSM710 confocal microscope at 63x magnification. The bars in the panels represent 10 µm. Nuclei and inclusion are indicated with N and Inc, respectively.

ZNF23 has been identified in a yeast two hybrid system with ACBD6 as bait (P. Hauser, E. Soupene and F. Kuypers, unpublished observations). ZNF23 is a KRAB_A containing zinc finger protein controlling cell cycle progression, and can act as a pro-apoptotic factor [27], [28]. Regardless of the precise role of the ZNF23 interaction with ACBD6, we decided to investigate its putative role in *Ct*-infected cells. As other ZNF proteins, ZNF23 is a nuclear protein and was detected in the nuclei of HeLa cells. Unexpectedly, it was also detected in the cytosol (Figure 4, panel A and Figure S6, panel A). The rabbit-raised antibody against ZNF23 detected a band of the correct molecular mass in protein extracts of human cells (Figure 4, panel A) and had no cross-reactivity with *Ct* proteins (Figure S1 and S2). As observed with ABCD6, ZNF23 disappeared from the cytosol and nucleus of cells infected with *Ct*, and was only detected in the inclusion lumen (Figure 6, panel B and Figure 7). These findings were confirmed in cells infected by three different *Ct* strains (strain D, Figure 7E; strain E, Figure 6B; strain L_2_, Figure 7 panel A to D), and with two different anti-ZNF23 antibodies. ZNF23 was detected in the inclusion with a rabbit antibody in cells infected with strain L_2_ (Figure 7, panel A to D) and strain E (Figure 6, panel B) and with a mouse antibody in cells infected with strain D (Figure 7, panel E). The mouse anti-ZNF23 antibody had no cross-reactivity with *Ct* proteins (Figure S2). As observed for ACLS3 and ACBD6, ZNF23 protein was detected in the lumen of the inclusion adjacent to the bacteria (Figure 7, panel D and E).

### Redistribution of ACBD6 and ZNF23 proteins in Ct-infected cells

**Figure 8:**
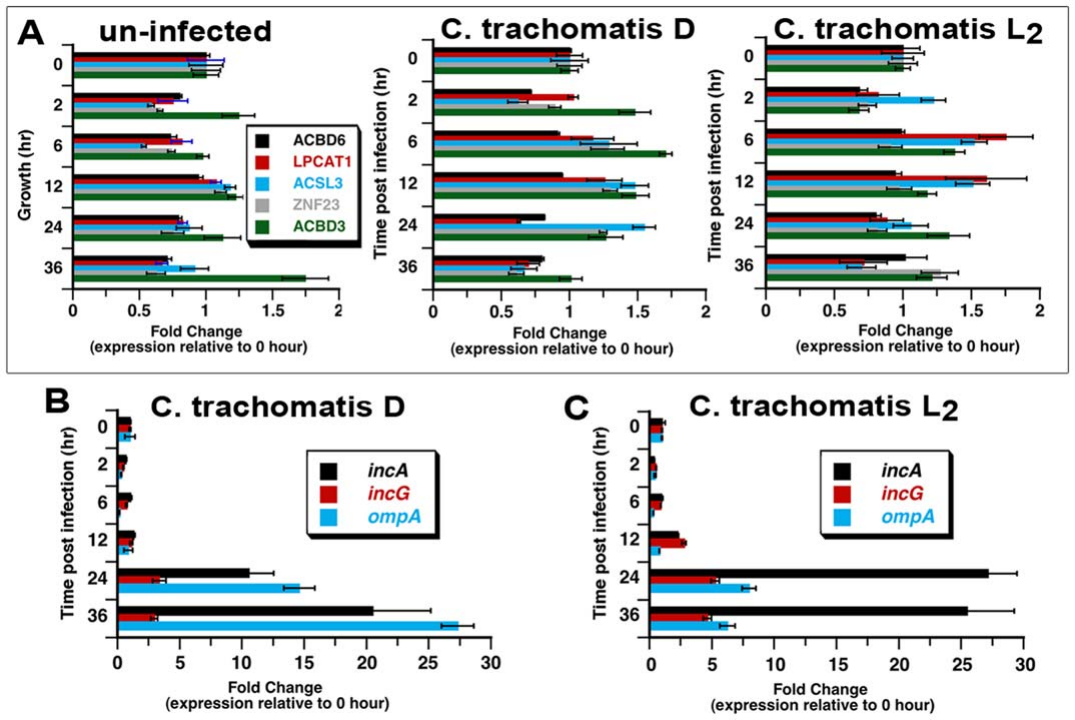
Expression of host proteins recruited to the inclusion is not altered in *Ct* infected cells. HeLa cells were grown in 24-well plates in cell culture medium without antibiotics and cycloheximide. Cells were infected at a MOI of 5 with *Ct* strain D (panel A. middle graph and panel B) and with L_2_ (panel A, right graph and panel C). Some cells were not infected (panel A, left graph). At the indicated time, total RNAs were isolated and cDNAs were synthesized with random primers. qPCR analysis was performed as described in the Method section. Expression levels of the human genes *ACBD3, ACBD6*, *ACSL3*, *LPCAT1* and *ZNF23* (panel A) were normalized to levels of *GAPDH* gene. In cells infected with *Ct* D (panel B) or with *Ct* L_2_ (panel C), *Ct* genes *incA*, *incG* and *ompA* were normalized to expression levels of the *euo* gene. For each gene, expression level ratio obtained at time point 0 hr was arbitrary set at a value of 1 and all other ratios were calculated relative to it. Note the difference of the x axis scale of the 3 plots in panel A compare to the graph in panel B and C.

As described above, ACBD6 and ZNF23 are recruited into the inclusion lumen, and are removed from the host compartment of the infected cells. To determine when these two events occurred and to assess if their disappearance was correlated to their accumulation in the inclusion, we established the expression and distribution profile of ACBD6 and ZNF23 in infected cells during the developmental cycle of *Ct.* At the mRNA level, we could not detect a significant change in expression of ABCD6 and ZNF23 from 0 to 36 hr post-infection for *Ct* strains D and L_2_ (Figure 8). In addition, mRNA levels of ACBD3, ACSL3 and LPCAT1 also remained fairly constant. As expected, expression of bacterial development markers, such as *incA*, *incG* and *ompA*, were induced several fold during growth of the bacteria. These results established that development of *Ct* had no effect on the regulation of the expression of these human genes and that disappearance of ACBD6 and ZNF23 was probably not the result of a transcriptional shut down mechanism.

**Figure 9:**
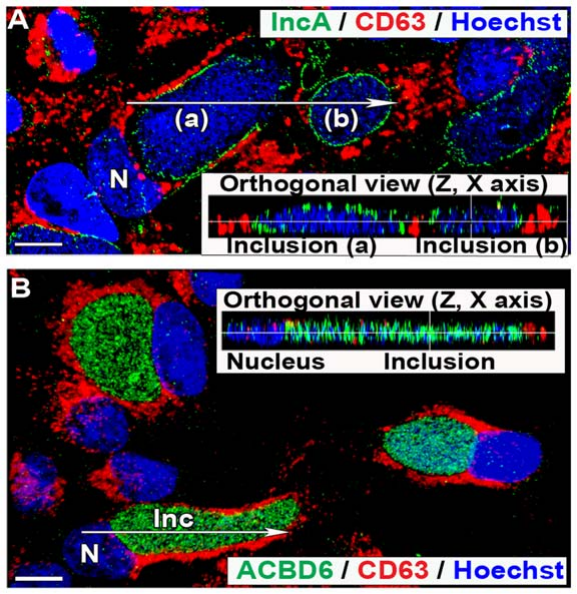
CD63 is not localized in the *Ct* inclusion of infected HEp-2 cells. HEp-2 cells were grown on coverslips and infected with *Ct* strain D at a MOI of 1 in cell culture medium without antibiotics and cycloheximide. Cells were fixed 44 hr post-infection. In panel A and B, CD63 protein was detected with a mouse monoclonal antibody (red). In panel A, the inclusion membrane was stained with a rabbit antibody against IncA (green), DNA was detected with the Hoechst dye (blue), and the merged image is shown. In panel B, the bacteria were only detected by Hoechst DNA staining and ACBD6 was detected with a rabbit antibody (green), and the merged image is shown. Signal distribution of the orthogonal (z, x) slice of deconvolved Z stack images, indicated by the arrow on the respective merged image, are displayed in the inset of each panels. Images were taken with a Zeiss LSM710 confocal microscope at 43x magnification. The bars in panels represent 10 µm. Nuclei and inclusion are indicated with N and Inc, respectively. Note that CD63 signal was not detected in the inclusions (panel A) and did not co-localize with ABCD6 protein, which was detected inside the inclusions (panel B).

At the protein level, ABCD6 was located inside inclusions as soon as these inclusions were detected at 12 hr (data not shown), and it was still present in the cytosol and nuclei of infected cells for the first 20 hours after infection (Figure 5, panel A to C). However, two hours later, the ACBD6 signal had disappeared from the host side of the inclusion membrane [Figure 5, panel C (20 hr) and panel D (22 hr)]. The ACBD6 signal persisted in the inclusions of *Ct* strain D late in development in HeLa cells [Figure 6, panel A (24 hr) and Figure 3, panel D (36 hr)] and in HEp-2 cells [Figure 3, panel H (36 hr) and Figure 9, panel B (44 hr)]. Similarly, ZNF23 protein was recruited into the inclusion as early in *Ct* development as ABCD6 was also detected (data not shown). However, whereas ABCD6 persisted in the host compartment for several more hours, ZNF23 signal disappeared from the cytosol and nuclei of infected cells as soon as the protein was mobilized into the inclusion [Figure S6, panel B (16 hr) and Figure 6, panel B (18 hr)].

## Discussion

The location of several host proteins is drastically altered during *Ct* development. Remarkably, enzymes located in different compartments of the host cell are recruited to the *Ct* inclusion. These enzymes are mobilized to the inclusion membrane (LPCAT1) or are translocated into the inclusion lumen (ACBD6, ACSL3, ZNF23). Although, some host proteins have been reported in the *Ct* inclusion membrane, none have consistently been shown to localize in the inclusion lumen [5]. The membrane protein CD59 was identified in the inclusion membrane facing the lumen, but not within the lumen [49]. A second protein, CD63, has been reported in the lumen of the *Ct* inclusion in HEp-2 cells by one group [50], [51]. CD63 was not detected in the inclusion lumen formed by *C. pneumoniae* in HEp-2 cells [52], and was not located with the bacterium in infected alveolar type II cells [53]. We were also not able to detect CD63 in the *Ct* inclusion lumen in HEp-2 cells (Figure 9). We have no explanation for the apparent detection of CD63 in *Ct* inclusions by Beatty *et* *al.* [50], [51]. To our knowledge, ACBD6, ACSL3, and ZNF23 represent the first examples of human proteins mobilized into the *Ct* inclusion lumen.

Branched-chain phosphatidylcholine is the second most abundant lipid of *Ct* membranes [22]. Straight-chain PC species produced in the ER membrane of the host cells are translocated to the membrane surrounding the inclusion. These host-PC species are de-acylated to lysoPC moieties in the inclusion membrane, probably by action of a host cPLA_2_, which is activated upon *Ct* infection [32], [54], [55]. *Ct*-produced branched-chain fatty acids are transferred onto host-lysoPC to generate the bacterial-PC species that are incorporated into *Ct* membranes [16], [17], [22]. We determined that human long-chain acyl-CoA synthetase member 3, ACSL3, is translocated into the inclusion lumen and that acyl-CoA:lysophosphatidylcholine acyltransferase member 1, LPCAT1, is recruited to the inclusion membrane. ACSL3 can generate the activated-fatty acid species necessary for the re-acylation reaction catalyzed by LPCAT1. ACSL3 and LPCAT1 specifically act toward synthesis of PC [37], [39], [56] and, therefore, likely also of the *Ct*-PC species.

We also found that the acyl-CoA binding protein ACBD6 was recruited into the inclusion lumen. Its role in PC metabolism is supported by its binding preference for long-chain fatty acids species abundant at the sn-2 position of PC [26]. The drug triacsin C, which inhibits some of the host acyl-CoA synthetase enzymes, has been shown to severely impair the growth and development of *Ct* [18]. Among the different members of the ACSL family, ACSL3 is the form most sensitive to triacsin C treatment, which could, in part, explain the noted effect on *Ct* growth and development. ACSL6, which was not mobilized into the inclusion, is not sensitive to the drug (reviewed in [25]).

ACSL3 and LPCAT1 enzymes also have the unusual property among membrane proteins to bind the lipid mono-layer surrounding lipid droplets [37], [39], [56]. In infected human cells, *Ct* proteins (Lda and IncA) are also associated with lipid droplets [14], [18]. Lipid droplets are thought to directly provide lipids to the bacteria and they appeared to be closely associated with the RBs inside the inclusion [4], [14], [18]. These lipid bodies can cross into the inclusion, without being absorbed by the phospholipid bilayer of the membrane [18]. Together with lipids that they contain, the host enzymes, in particular ACSL3, that are associated with these lipid bodies could then translocate into the inclusion lumen. In that context, these lipid bodies could represent a major support mechanism for the lipid metabolism of *Ct*.

ZNF23 is a member of the C_2_H_2_-type sub-family of the large zinc finger family. ZNF23 contains 17 repeats of the Cys_2_-His_2_ motif and belongs to the class of ZF proteins with Krüppel-associated box (KRAB) type A [27], [28]. These nuclear proteins are transcriptional factors usually responsible for repression by recruitment of other protein(s). ZNF23 is a pro-apoptotic factor that acts as a repressor of cell division [27], [28]. The role of ZNF23 in lipid metabolism is unknown but preliminary results indicate that it can interact with ACBD6 protein in a yeast two-hybrid system (P. Hauser, E. Soupene and F. Kuypers, unpublished observations). ACBD6 and ZNF23 are located in the cytosol and nucleus of cells. In cells infected by *Ct* for more than 20 hr, we found that these two proteins were no longer present in host cell nuclei or cytoplasm but were located in the inclusion lumen. However, the depletion observed for ACBD6 and ZNF23 was not detected for ACSL3, which was also recruited into the inclusion lumen, and there were no detectable changes in mRNA levels for any of the enzymes we tested. Thus, ACBD6 and ZNF23 appear to be the target of an, as yet, unidentified removal mechanism that takes place when inclusions have started to fuse and enlarge. This mechanism(s) did not appear to affect the intracellular location of any of the other proteins we studied (ACSL3, ACSL6, ACBD3, CD63, LPCAT1). Sequestration into the inclusion would protect ACBD6 and ZNF23 from degradation, which likely occurs in the cytosol of the host-infected cell.

Human infected cells are protected against stimuli inducing the mitochondrion-dependent cell death program [4], [31], [33], [35]. *Ct*-infected cells activate anti-apoptotic signaling pathways, such as the Raf/MEK/ERK survival pathway, recruit host death receptors to the inclusion membrane, and several pro-apoptotic host proteins are targeted for degradation by *Ct* secreted proteases [32]–[34], [36], [57]–[59]. ZNF23, which we discovered was also recruited into the inclusion lumen, has been shown to down-regulate the level of the Bcl-X_L_ protein in some cancer cells [27]. Bcl-X_L_ is a member of the BCL-2 family. It acts against the pro-apoptotic function of BAX-BAK complex and prevents cytochrome c-released (review in [4], [31], [60]). Activation of BAX-BAK, by BH3-containing proteins such as BAD, is prevented in *Ct*-infected cells [36]. Early in the developmental process, *Ct* CPAF protease is secreted into the host cytosol where it targets BAD and other human BH3-proteins for degradation [33], [34], [57], [61]. Moreover, the phosphorylated form of BAD is sequestered away from the mitochondria membrane by its association with the host protein 14-3-3ß at the inclusion membrane. [36]. Protein 14-3-3ß is recruited by means of its association with the phosphorylated form of the bacterial IncG protein [62]. IncG, produced by *Ct* inside the inclusion, is imported to the membrane of the inclusion where it is phosphorylated by a host tyrosine kinase [42], [62]. Although, not completely understood, a regulated and coordinated mechanism is taking place on each side of the inclusion membrane to prevent the death of the host cells. Our findings suggest that in addition to the sequestration and inactivation of the ‘pro-pro-apoptotic’ BAD protein, the complete translocation of ZNF23 into the inclusion lumen, perhaps through its interaction with ACBD6 in complex with lipid bodies engulfed into the inclusion, would protect the infected cells from the ‘anti-anti-pro-apoptotic’ effect of ZNF23.

Our results provide further evidence that via protection inside the parasitophorous vacuole, *Ct* can disrupt host cellular pathways, such as lipid body trafficking and initiation of programmed cell-death, by secretion of bacterial proteins and by mobilization of specific host enzymes into the inclusion lumen.

## Materials and Methods

### Cell culture and infection

HeLa229 cells [63], obtained from ATCC (CCL-2.1) were grown in minimal essential medium with glutamine (MEM alpha, Invitrogen, Carlsbad, CA) containing 10% fetal bovine serum (UCSF Cell Culture Facility, San Francisco, CA). HEp-2 cells [50], [51], obtained from ATCC (CCL-23) were maintained in low-dextrose MEM with glutamine (DMEM, Invitrogen). For microscopy studies, cells were grown, transfected and infected on 12-mm round coverslips (Electron Microscopy Sciences, Inc., Hatfield, PA) in 24-well plates (E&K Scientific, Santa Clara, CA). Cells grown to 70% confluence were infected with *Ct* strains D, E or L_2_ at a multiplicity of infection (MOI) of 1 to 5, as indicated in the legend of the figures and as previously described [63]. After 1 hr, the inocula were aspirated and fresh growth medium supplemented with 40 µM gentamicin (MP Biomedicals, Solon, OH) was added to the infected cells. At the indicated times, medium was aspirated, cells were washed twice with PBS and were fixed with ice-cold methanol for 10 minutes. After aspiration cells were washed twice with PBS and kept at 4°C until use.

### Immunochemical detection

Fixed cells were incubated in a blocking solution (PBS with 0.1% Tween-20 and 5% goat serum) over-night at 4°C. Incubations with primary antibodies were performed in 400 µl PBS with 0.1% Tween-20 and 1% BSA, for 3 to 4 hr at room temperature on a rocking platform. To detect bacteria, a *Ct*-specific lipopolysaccharide (LPS) monoclonal antibody (MAb) (Virostat, Portland, ME) at a 1/1,000 dilution and a *Ct*-specific IncA polyclonal antibody (generous gift from Ted Hackstadt to DD) at a 1/200 dilution were used. Antibodies against human proteins included a mouse monoclonal against ACSL3 (clone H9, Santa Cruz Biotechnology, Inc.), a MAb against ACBD3 (clone 2H2, Abnova, Jhongli, Taiwan), a MAb against CD63 (clone MX-49.129.5, Santa Cruz Biotechnology, Inc. Santa Cruz, CA), a mouse polyclonal against ZNF23 (ab68252, Abcam Inc., Cambridge, UK), an affinity-isolated rabbit polyclonal against ZNF23 (SAB4503115, Sigma-Aldrich, St Louis, MI), and affinity-purified custom-made rabbit polyclonal against ACBD6 [26] and ACSL6 [64]. All antibodies were used at a dilution of 1/500. Conjugated secondary antibodies were incubated protected from light for 1 to 2 hr under the same condition. Cells were washed three times in PBS, and DNA was revealed by staining with 200 µl of 0.5 µg/ml Hoechst 33258 dye (Invitrogen, Carlsbad, CA) in PBS for 10 min. Dye was removed by two washes with PBS.

### Microscope image acquisition

Coverslips were mounted on glass slides embedded in FluorSave Reagent (EMD Biosciences, Darmstadt, Germany). Microscopy analysis was performed with a Zeiss LSM 710 confocal inverted microscope at room temperature. Magnifications and scale bars are indicated in the legend of each figure. Image processing, deconvolution, 3D reconstruction and colocalization analysis were performed with Huygens Essential and Bitplane Imaris Suite package of Scientific Volume Imaging (Hilversum, The Netherlands).

### Proteins sample preparation and cell fractionation

For isolation of nuclei, HeLa cells were grown to near confluence in a T75 flask and harvested by trypsin treatment. The cell pellet was kept on ice and was washed once in ice-cold hypotonic buffer containing 10 mM HEPES pH 7.9, 1.5 mM MgCl_2_, 10 mM KCl, 0.5 mM DTT and 0.2 mM PMSF. Cells were suspended in 5 volumes (≈ 1 ml) of the same buffer and allowed to swell for 20 min on ice. The cells were transferred in a Dounce homogenizer and homogenized by 10 to 15 strokes. Complete lysis of the cells was determined by light microscopy. The broken cell suspension was centrifuged at 6,000 rpm for 5 min. The supernatant was separated from the pellet, representing the crude cytosolic fraction. The pellet was gently washed with hypotonic buffer, and the presence of intact nuclei was verified by staining with trypan blue observed under light microscopy. The nuclei pellet was suspended in a small volume of buffer (≈ 100 µl). Protein concentration in the supernatant and nuclei was determined with the colorimetric RC-DC Protein assay (Bio-Rad, Hercules, CA) using BSA as standard. The nuclei preparation were lysed and dissolved by boiling in the SDS-PAGE loading buffer for 5 min. Proteins samples were separated under denaturing condition on 4–16% gradient polyacrylamide gel (Bio-Rad). Protein samples of EBs were prepared by boiling 50 µl of a frozen stock of 5x10^8^ per ml of purified EBs of *Ct* strain D or L_2_ with 50 µl of SDS-PAGE loading buffer 2x for 5 min and by vigorous vortexing. Samples were centrifuged for 30 s at full-speed (14,000 g) to remove insoluble debris, and 10 µl were loaded on a 4–16% gradient polyacrylamide gel (Bio-Rad). Following electrophoresis, proteins were transferred on PVDF membrane.

### Western blot immunodetection

PVDF membrane were blocked overnight at 4°C in TBS with 0.05% Tween-20 and 5 % non-fat milk. Primary antibodies (mouse anti-ACSL3, mouse anti-HSP60, rabbit anti-ACBD6, rabbit anti-ANKRD7, and rabbit anti-ZNF23) were diluted 1/2,500 in TBS with 0.05% Tween-20 and 1 % milk and incubated 2 to 4 hr at room temperature on a rocking platform. Peroxidase-conjugated secondary antibodies (goat anti-mouse and goat anti-rabbit) (Bio-Rad), diluted 1/1,000 in TBS with 0.05% Tween-20 and 1 % milk, were incubated 1 to 2 hr under the same condition. Detection was performed with SuperSignal West Pico Chemiluminescent kit (Thermo Fisher Scientific Inc., Rockford, IL).

### DNA manipulation and transfection

Full-length LPCAT1 cDNA [30] and ACSL6 cDNA [64], [65] were cloned into the pAcGFP1 vector (Clontech Laboratories, Mountain View, CA) to generate a fusion of the GFP protein to their amino-terminal extremity. Briefly, PCR cloning was performed with High-Fidelity Expand Taq DNA polymerase (Roche Applied Science, Indianapolis, IN) using the plasmid pFK182 as template, and the following primer pair (5′-CTAGAGCTCAGACACAGGAGATCCTGAGGATAC-3′; 5′-CTAGGATCCTCACATGGAGATTGAGTAAAGCTCTTC-3′).

The PCR product was cloned with Zero-Blunt PCR cloning kit (Invitrogen) and was sequenced to comfirm the absence of base error. The vector was cloned in-frame at the *Eco*ICRI/*Bam*HI restriction sites of pAcGFP1 to yield plasmid pFK338. A similar cloning strategy was used for LPCAT1. The PCR product was obtained with template pFK189 and the following primer pair (5′-TTGATATCCTAGCGCCGCCCCCTCCTCC-3′; 5′-ATGGATCCCTAGTCCGCTTTCTTACAAGAATTC-3′), to yield plasmid pFK642. DNA transfections of HeLa cells grown on coverslips in 24-well plates were performed with NanoJuice transfection reagent (EMD Bioscience, Darmstadt, Germany) using 0.8 µg of circular plasmid DNA, a DNA to Core ratio of 1 and, a DNA to Booster ratio of 4. Cells were fixed or infected with strains D or L_2_ after 24 hr.

### Reverse transcription and real-time PCR

For gene expression analysis, HeLa cells were infected in duplicate in 48-well plates at an MOI of 5. Total RNA was extracted at 0, 2, 6, 12, 24 and 36 hr postinfection using RNeasy MINI kit (Qiagen, Valencia, CA) following the manufacturer's instructions with an on-column DNase treatment (Qiagen, Valencia, CA). Following elution off the column, trace amounts of DNA in the RNA preparation was further removed by treatment with *TurboDNase* (Ambion, Austin, TX). DNA-free RNAs were extracted with phenol-chloroform and concentrated by ethanol precipitation. No-RT control PCR reactions were performed with iTAQ SYBR Green® Supermix (Bio-Rad, Hercules, CA) using a human *ACTB* primer pair or a *Ct ompA* primer pair on an ABI7000 instrument. RNA samples were considered DNA-free when no signal was detected at C-threshold values less than 35 for a 40 cycle amplification program.

Bacterial gene expression was determined with *ompA*, *incA*, *incG* and *euo* gene-specific PrimeTime qPCR probes conjugated with FAM fluorescent dye and with Iowa Black®FQ and Internal ZEN quenchers (IDT DNA Technologies, Coralville, IO). Sequence of primer (fwd/rev) and probe sets were as follow: *ompA* [(5′-TTC TAT GGG AAG GTT TCG GC-3′/5′-CAC GGT CGA AAA CAA AGT CAC-′3) and probe 5′-/56-FAM/AGA TCC TTG/ZEN/CGA TCC TTG CAC CA/3IABkFQ/-3′]; *incA* [(5′-TTT TAG CTC TTT TGG GAC ATC TTG-3′/5′-TGC TAA TGA GGT AAT GAA TAG GGC-3′) and probe 5′-/56-FAM/TGG CTT TCT/ZEN/GAT CGC TCC ACA CA/3IABkFQ/-3′]; *incG* [(5′-ACG AAA TGC TTA CAA ACG GC-3′/5′-AGT GCC ACT AAA CAG TAC CG-3′) and probe 5′-/56-FAM/TTC CTG AAG/ZEN/CAA ACA CCG CAA ACA A/3IABkFQ/-3′]; *euo* [(5′-TTA TTC CGT GGG ACA AGT GG-3′/5′-TGA ATG ACC CAA GCA GAT CC-3′) and probe 5′-/56-FAM/ATT GGT GCT/ZEN/ATG AAA GGA GAG CGT CG/3IABkFQ/-3′]. cDNA was generated from 2 µg of total RNA using random primers and AffinityScript QPCR cDNA Synthesis kit (Agilent Technologies Inc., Santa Clara, CA) in a 20 µl reaction, according to manufacturer's instructions. Real-time PCR reactions were performed with 0.25 µl of cDNA per reaction and 500 nM of gene-specific qPCR probe, using Brilliant II QPCR Master Mix with ROX (Agilent Technologies Inc., Santa Clara, CA). Quadruplicates reactions were performed in 5 µl volume in 384-well plate format and were run on a ABI7900HT instrument (Applied Biosystems, Foster City, CA).

Human gene expression was determined with *ACBD3, ACBD6*, *ACSL3*, *LPCAT1*, *ZNF23* and GAPDH gene-specific RT^2^ qPCR Primers (Qiagen, Valencia, CA). Real-time PCR reactions were performed with 0.5 µl of cDNA per reaction and 400 nM gene-specific RT^2^ qPCR Primer, using iTAQ SYBR Green® Supermix with ROX (Bio-Rad, Hercules, CA). Reactions were performed in triplicate in 13 µl volume in 96-well plate format and were run on a ABI7000 instrument (Applied Biosystems, Foster City, CA).

Expression levels of *Ct incA*, *incG* and *ompA* genes were normalized to the values obtained for the *euo* gene [42]. Expression of human *ACBD3, ACBD6*, *ACSL3*, *LPCAT1* and *ZNF23* genes were normalized to the values obtained for the *GAPDH* gene. For each gene, normalized value obtained at time 0 was used as reference. The ΔΔCt method was used to determine the expression level values of bacterial and human genes in infected cells from 2 to 36 hr relative to 0 hr.

## Supporting Information

Figure S1 **Lack of anti-human protein antibody reactivity against** ***Ct*** **proteins analyzed** **by Western blot.** Proteins were separated under denaturing condition in presence of SDS, and result of a coomassie blue staining is shown on the right. Following electrophoresis, the proteins were transferred onto a PVDF membrane. After transfer, the membrane was cut as indicated on panel A and B. After blocking, each portion was incubated with primary antibodies against the human proteins ACBD6, ACSL3 or ZNF23. *Ct* protein HSP60 and host GAPDH were used as controls. Detection was performed with SuperSignal West Pico Chemiluminescent kit (Thermo Scientific). As indicated, the portions of the membranes were exposed to the same films for the same period of time. Panel A. Protein extracts of *Ct* strain D and L_2_, prepared as described in the Method section, were boiled in presence of SDS-PAGE loading buffer and approximatively 10 µg was loaded in each lane of a gradient polyacrylamide gel. Panel B. Mouse Mc Coy cells were infected with Ct D at a MOI of 1. Proteins from uninfected cells (-) and from cells infected for 24 hrs (+) were isolated and analyzed as described in panel A. As shown, even under long exposure conditions, which resulted in a very strong signal for the bacterial protein HSP60 and host GAPDH no signal was detected in EBs and in Ct-infected McCoy cells with the antibodies used against the human proteins.
(TIF)

Figure S2 **Lack of** **anti-human protein antibody reactivity against** ***Ct*** **proteins as shown by immuno-histochemistry.** Mouse McCoy cells were grown on coverslips in cell culture medium without antibiotics and cycloheximide. At near confluence, cells were fixed (uninfected, left panels) or were infected with *Ct* strain D at a MOI of 1 and were fixed 36 hr post-infection. DNA was stained with the Hoechst 33258 dye (blue). Human ACSL3 and ZNF23 were detected with mouse antibody stained with a Cy^TM^3-conjugated anti-mouse antibody (red) and bacterial MOMP protein was detected with a rabbit antibody stained with an AlexaFluor®488-conjugated anti-rabbit antibody (green). Human ACBD6 and ZNF23 were detected with rabbit antibody stained with an AlexaFluor®488-conjugated anti-rabbit antibody (green) and bacterial LPS was detected with a mouse monoclonal antibody stained with Cy^TM^3-conjugated anti-mouse antibody (red). Merged images are shown on the right panel of each row. Images were taken with a Zeiss LSM710 confocal microscope at 40x magnification. The bars in panels represent 10 µm. Note that in each row, a cropped snap shot of panel 2 is shown in panel 3, 4, 5, and 6. None of the anti-human antibody reacted with antigen in the inclusion.
(TIF)

Figure S3 **GFP-ACSL6 protein is not detected in C** ***t*** **inclusion.** HeLa cells were grown on coverslips and were transfected with a DNA construct expressing a GFP-ACSL6 fusion. After 24 hr, cells were infected in cell culture medium without antibiotics and cycloheximide with *Ct* strain L_2_ at a MOI of 1. Cells were fixed 24 hr post-infection. Bacterial LPS was detected with a mouse monoclonal antibody, which was stained with Cy^TM^3-conjugated anti-mouse antibody (red), DNA was stained with the Hoechst 33258 dye (blue) and merged images with LPS signal (middle panel) and with LPS and GFP (right panel) are shown. Images were taken with a Zeiss LSM710 confocal microscope at 63x magnification. The bar in the panel represents 10 µm. Nuclei and inclusion are indicated with N and Inc, respectively. ACSL6 is not recruited to the inclusion.
(TIF)

Figure S4 **Competition of the immuno-histological detection of ACBD6** **by an ACBD6 peptide.** HeLa cells grown on coverslips in cell culture medium without antibiotics and cycloheximide. At near confluence, some cells were fixed (images shown) or were infected with *Ct* strain D at a MOI of 1 and fixed 36 hr post-infection (data presented in the inset). Affinity-purified polyclonal anti-ACBD6 antibody was stained with an anti-rabbit antibody labeled with AlexaFluor®488 (green). DNA was stained with Hoechst 33258 dye (blue). For peptide treatment, 40 nmole of the synthetic antigenic ACBD6 peptide was added during the incubation with the primary antibody (anti-ACBD6 antibody). Images were taken with a Zeiss LSM710 confocal microscope at 40x magnification using the same exposure settings for the un-treated and treated coverslips. The bars in panels represent 10 µm. Note that whereas Hoechst dye's staining was of similar signal intensity in un-treated and treated samples, signal intensity for ACBD6 of the treated cells was visibly weaker in presence of the peptide. The same treatment was performed on infected cells, and the result of a semi-quantitative analysis is shown in the inset. To estimate the decrease in signal intensity observed for staining of ACBD6 in *Ct* inclusions in presence of the peptide, signal intensity in the nuclei of the infected cells was determined, and it was used to normalize the intensity values obtained in the inclusion. The signal intensity of at least 15 different cells per condition was determined. The signal intensity of Hoechst staining across the nucleus was expressed relative to the length of the section in µm and the assumption was made that this value was not affected by the presence or absence of the ACBD6 peptide. The signal intensities of ACBD6 staining per µm were determined across the inclusion of the same cells. The ratio of these values obtained for the untreated coverslip was arbitrary set at 1 and the ratio of the values for the treated sample was calculated from it.
(TIF)

Figure S5 **Golgi-associated ACBD3 is not recruited to the** ***Ct*** **inclusion.** HeLa cells grown on coverslips in cell culture medium without antibiotics and cycloheximide. At near confluence, some cells were fixed (panel A) or were infected with *Ct* strain L_2_ at a MOI of 1 and were fixed 36 hr post-infection (panel B). Human ABCD3 protein was detected with a mouse monoclonal antibody stained with a Cy^TM^3-conjugated anti-mouse antibody (red). In infected cells, bacterial MOMP protein was detected with a rabbit antibody, which was stained with an AlexaFluor®488-conjugated anti-rabbit antibody (green). DNA was stained with the Hoechst 33258 dye (blue). Merged images are shown. Images were taken with a Zeiss LSM710 confocal microscope at 40x magnification. The bars in panels represent 10 µm. On panel B, representative inclusions and nuclei are indicated with Inc and capital N, respectively. ACBD3 protein is not recruited into the inclusion.
(TIF)

Figure S6 **Redistribution of ZNF23 from the nucleus and cytosol into the inclusion lumen during** ***Ct*** **development.** HeLa cells were grown on coverslips in cell culture medium without antibiotics and cycloheximide. At near confluence, some cells were fixed (panel A, 0 hr) or were infected with *Ct* strain E at a MOI of 1 and were fixed 16 hr post-infection (panel B). Human ZNF23 protein was detected with a rabbit antibody stained with an AlexaFluor®488-conjugated anti-rabbit antibody (green). DNA was stained with the Hoechst 33258 dye (blue). Images were taken with a Zeiss LSM710 confocal microscope at a magnification of 40x in panel A and of 63x in panel B. The bars in the panels represent 20 µm. Note that the bacteria were only detected by staining their DNA with the Hoechst dye. The results obtained for staining of ZNF23 and the merged image obtained with stained DNA are shown. On panel B, an inclusion (Inc) and a nucleus are indicated by an arrow and with a capital N, respectively. In un-infected cells (panel A), ZNF23 is located in the cytosol and nucleus. In infected cells, ZNF23 has been recruited into the inclusions and is no longer detected in the host cell.
(TIF)
