# Supplementary figures and images for: Eukaryotic Protein Recruitment into the Chlamydia Inclusion: Implications for Survival and Growth
Source: PLoS One. 2012 May 9;7(5):e36843. doi: 10.1371/journal.pone.0036843 (PMC3348897; doi:10.1371/journal.pone.0036843)

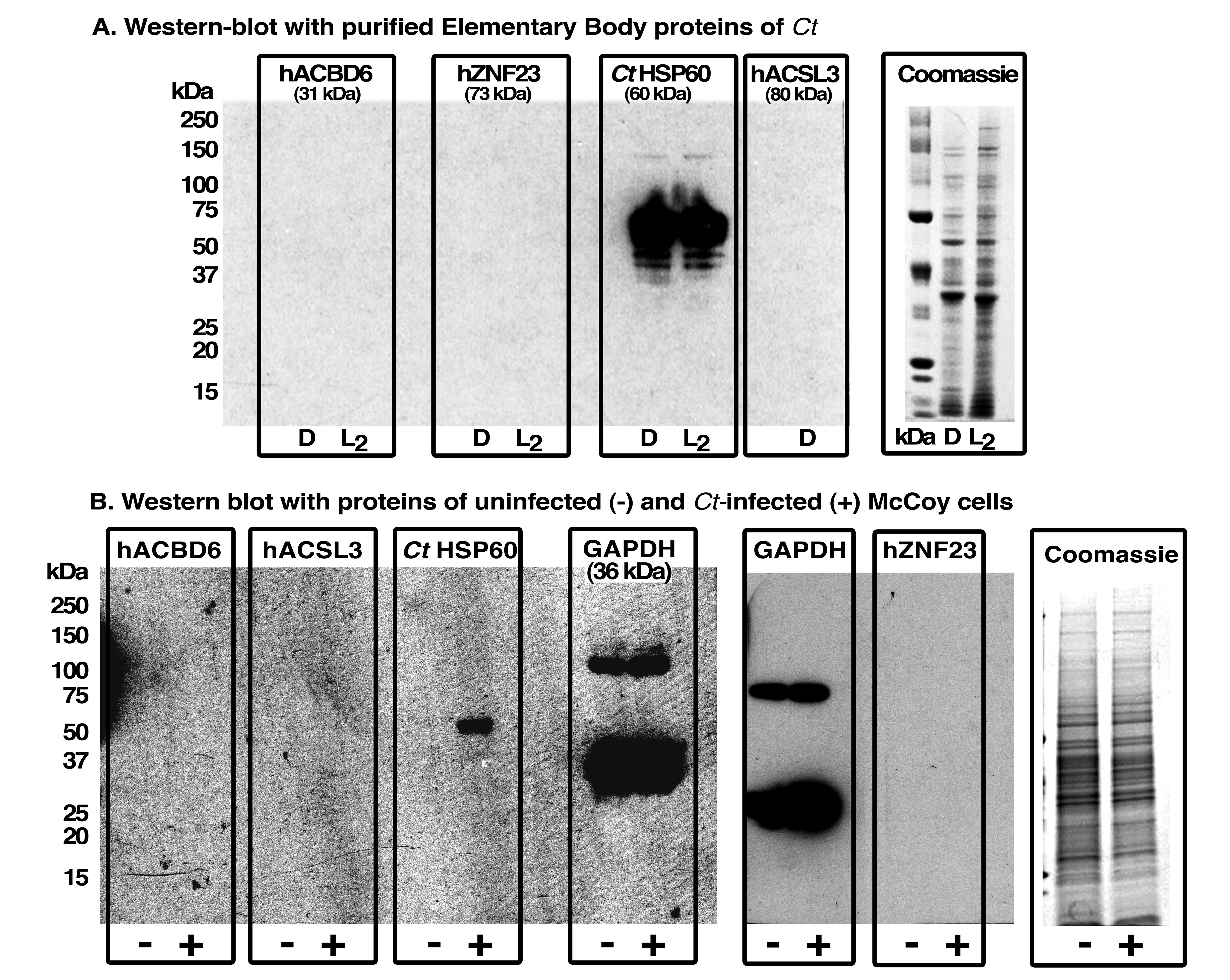

Supplement: Figure S1 — Lack of anti-human protein antibody reactivity against Ct proteins analyzed by Western blot. Proteins were separated under denaturing condition in presence of SDS, and result of a coomassie blue staining is shown on the right. Following electrophoresis, the proteins were transferred onto a PVDF membrane. After transfer, the membrane was cut as indicated on panel A and B. After blocking, each portion was incubated with primary antibodies against the human proteins ACBD6, ACSL3 or ZNF23. Ct protein HSP60 and host GAPDH were used as controls. Detection was performed with SuperSignal West Pico Chemiluminescent kit (Thermo Scientific). As indicated, the portions of the membranes were exposed to the same films for the same period of time. Panel A. Protein extracts of Ct strain D and L2, prepared as described in the Method section, were boiled in presence of SDS-PAGE loading buffer and approximatively 10 µg was loaded in each lane of a gradient polyacrylamide gel. Panel B. Mouse Mc Coy cells were infected with Ct D at a MOI of 1. Proteins from uninfected cells (-) and from cells infected for 24 hrs (+) were isolated and analyzed as described in panel A. As shown, even under long exposure conditions, which resulted in a very strong signal for the bacterial protein HSP60 and host GAPDH no signal was detected in EBs and in Ct-infected McCoy cells with the antibodies used against the human proteins. (TIF) [file pone.0036843.s001.tif]

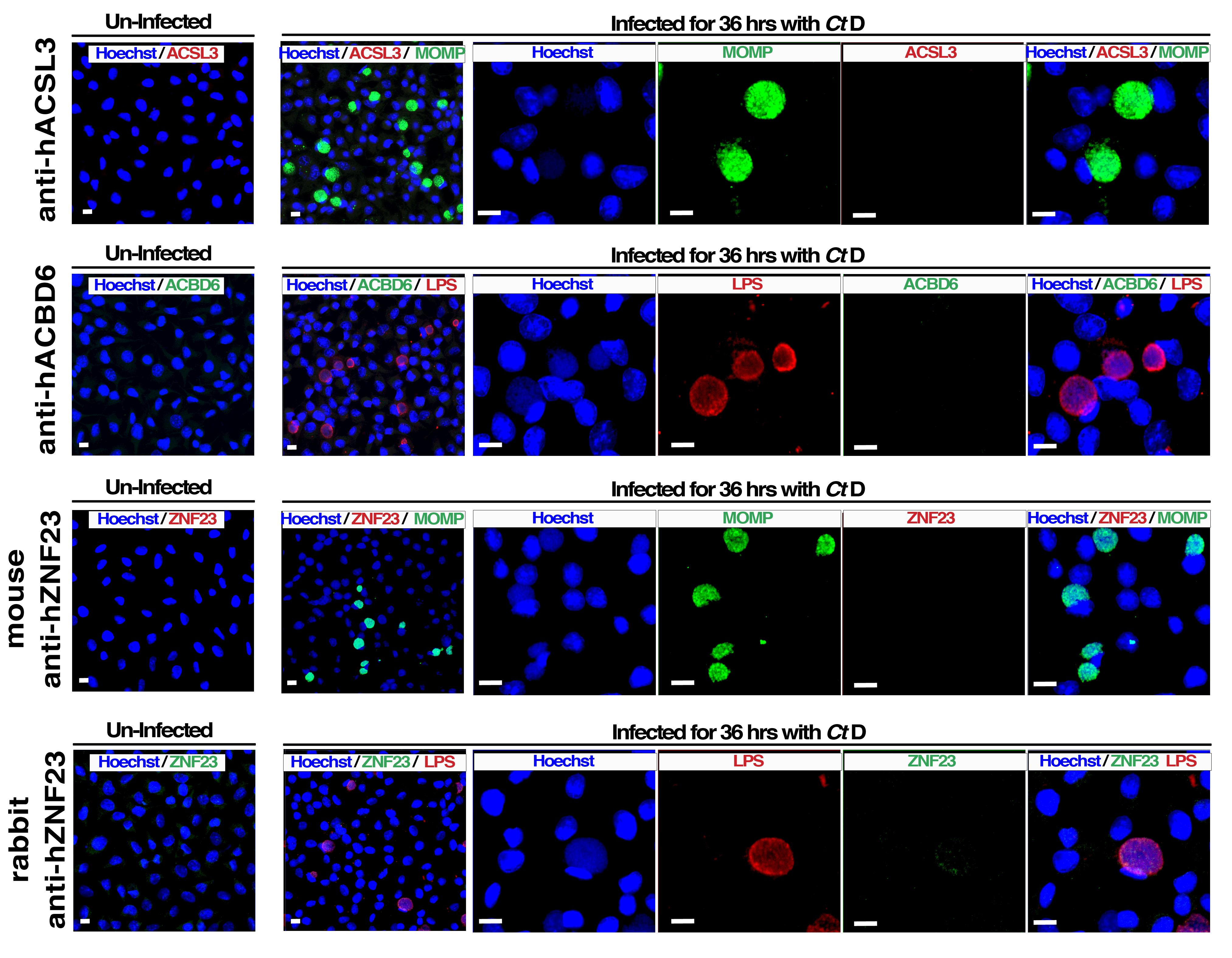

Supplement: Figure S2 — Lack of anti-human protein antibody reactivity against Ct proteins as shown by immuno-histochemistry. Mouse McCoy cells were grown on coverslips in cell culture medium without antibiotics and cycloheximide. At near confluence, cells were fixed (uninfected, left panels) or were infected with Ct strain D at a MOI of 1 and were fixed 36 hr post-infection. DNA was stained with the Hoechst 33258 dye (blue). Human ACSL3 and ZNF23 were detected with mouse antibody stained with a CyTM3-conjugated anti-mouse antibody (red) and bacterial MOMP protein was detected with a rabbit antibody stained with an AlexaFluor®488-conjugated anti-rabbit antibody (green). Human ACBD6 and ZNF23 were detected with rabbit antibody stained with an AlexaFluor®488-conjugated anti-rabbit antibody (green) and bacterial LPS was detected with a mouse monoclonal antibody stained with CyTM3-conjugated anti-mouse antibody (red). Merged images are shown on the right panel of each row. Images were taken with a Zeiss LSM710 confocal microscope at 40x magnification. The bars in panels represent 10 µm. Note that in each row, a cropped snap shot of panel 2 is shown in panel 3, 4, 5, and 6. None of the anti-human antibody reacted with antigen in the inclusion. (TIF) [file pone.0036843.s002.tif]

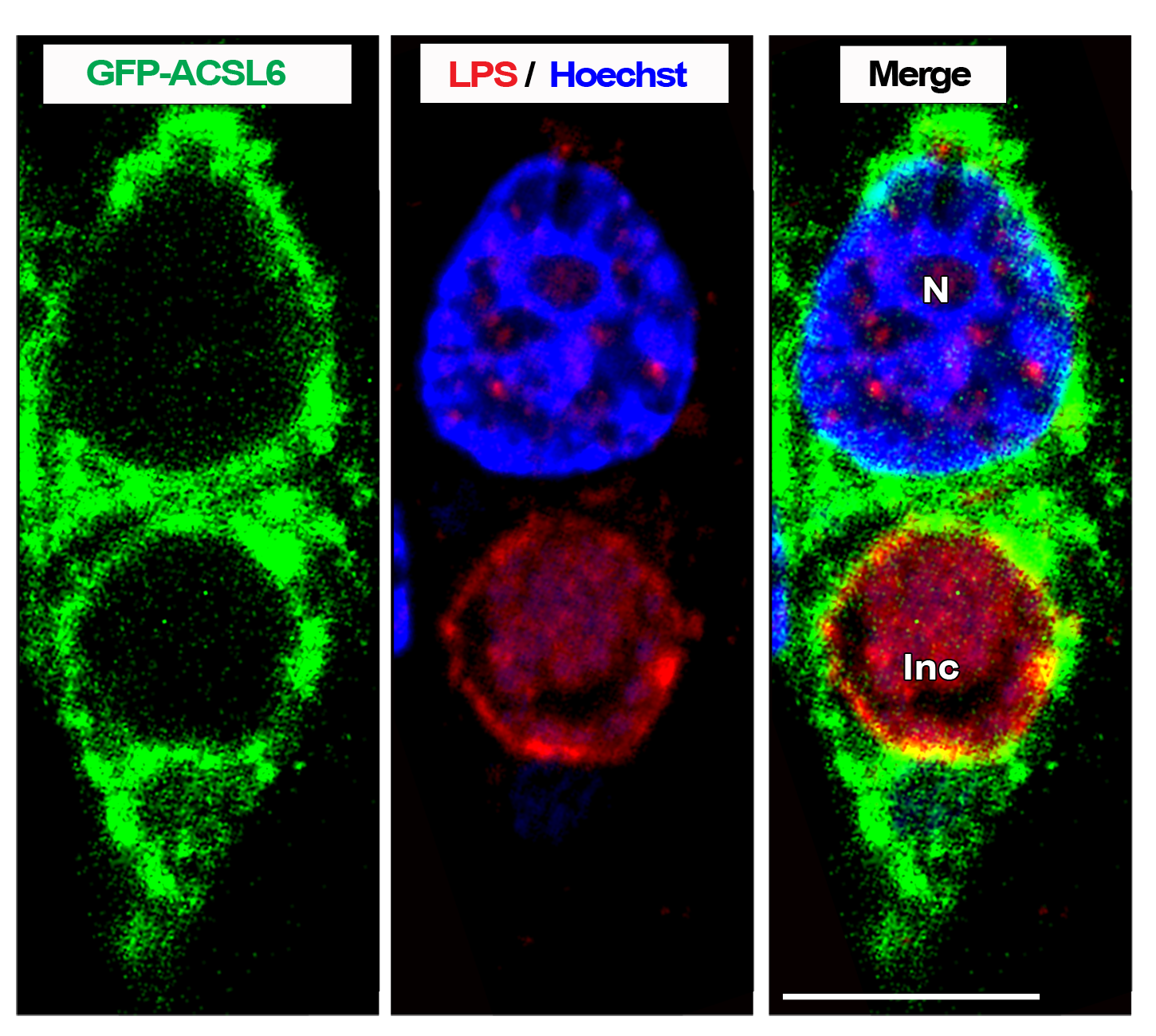

Supplement: Figure S3 — GFP-ACSL6 protein is not detected in C t inclusion. HeLa cells were grown on coverslips and were transfected with a DNA construct expressing a GFP-ACSL6 fusion. After 24 hr, cells were infected in cell culture medium without antibiotics and cycloheximide with Ct strain L2 at a MOI of 1. Cells were fixed 24 hr post-infection. Bacterial LPS was detected with a mouse monoclonal antibody, which was stained with CyTM3-conjugated anti-mouse antibody (red), DNA was stained with the Hoechst 33258 dye (blue) and merged images with LPS signal (middle panel) and with LPS and GFP (right panel) are shown. Images were taken with a Zeiss LSM710 confocal microscope at 63x magnification. The bar in the panel represents 10 µm. Nuclei and inclusion are indicated with N and Inc, respectively. ACSL6 is not recruited to the inclusion. (TIF) [file pone.0036843.s003.tif]

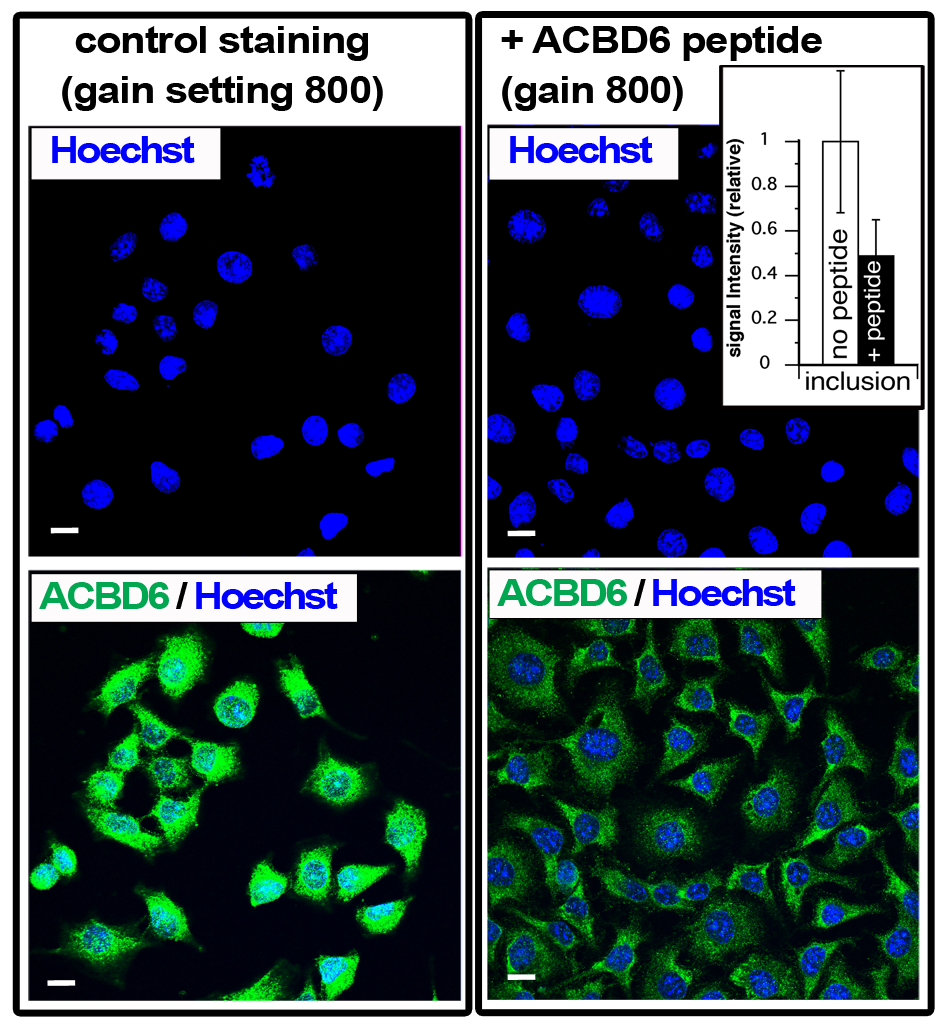

Supplement: Figure S4 — Competition of the immuno-histological detection of ACBD6 by an ACBD6 peptide. HeLa cells grown on coverslips in cell culture medium without antibiotics and cycloheximide. At near confluence, some cells were fixed (images shown) or were infected with Ct strain D at a MOI of 1 and fixed 36 hr post-infection (data presented in the inset). Affinity-purified polyclonal anti-ACBD6 antibody was stained with an anti-rabbit antibody labeled with AlexaFluor®488 (green). DNA was stained with Hoechst 33258 dye (blue). For peptide treatment, 40 nmole of the synthetic antigenic ACBD6 peptide was added during the incubation with the primary antibody (anti-ACBD6 antibody). Images were taken with a Zeiss LSM710 confocal microscope at 40x magnification using the same exposure settings for the un-treated and treated coverslips. The bars in panels represent 10 µm. Note that whereas Hoechst dye's staining was of similar signal intensity in un-treated and treated samples, signal intensity for ACBD6 of the treated cells was visibly weaker in presence of the peptide. The same treatment was performed on infected cells, and the result of a semi-quantitative analysis is shown in the inset. To estimate the decrease in signal intensity observed for staining of ACBD6 in Ct inclusions in presence of the peptide, signal intensity in the nuclei of the infected cells was determined, and it was used to normalize the intensity values obtained in the inclusion. The signal intensity of at least 15 different cells per condition was determined. The signal intensity of Hoechst staining across the nucleus was expressed relative to the length of the section in µm and the assumption was made that this value was not affected by the presence or absence of the ACBD6 peptide. The signal intensities of ACBD6 staining per µm were determined across the inclusion of the same cells. The ratio of these values obtained for the untreated coverslip was arbitrary set at 1 and the ratio of the values for the treated sample [file pone.0036843.s004.tif]

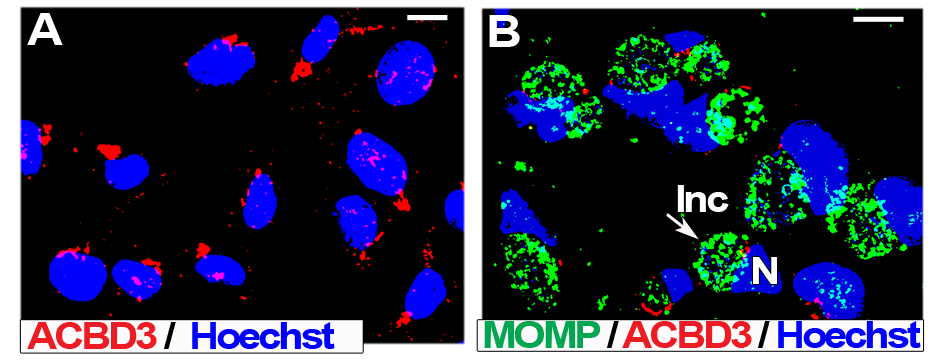

Supplement: Figure S5 — Golgi-associated ACBD3 is not recruited to the Ct inclusion. HeLa cells grown on coverslips in cell culture medium without antibiotics and cycloheximide. At near confluence, some cells were fixed (panel A) or were infected with Ct strain L2 at a MOI of 1 and were fixed 36 hr post-infection (panel B). Human ABCD3 protein was detected with a mouse monoclonal antibody stained with a CyTM3-conjugated anti-mouse antibody (red). In infected cells, bacterial MOMP protein was detected with a rabbit antibody, which was stained with an AlexaFluor®488-conjugated anti-rabbit antibody (green). DNA was stained with the Hoechst 33258 dye (blue). Merged images are shown. Images were taken with a Zeiss LSM710 confocal microscope at 40x magnification. The bars in panels represent 10 µm. On panel B, representative inclusions and nuclei are indicated with Inc and capital N, respectively. ACBD3 protein is not recruited into the inclusion. (TIF) [file pone.0036843.s005.tif]

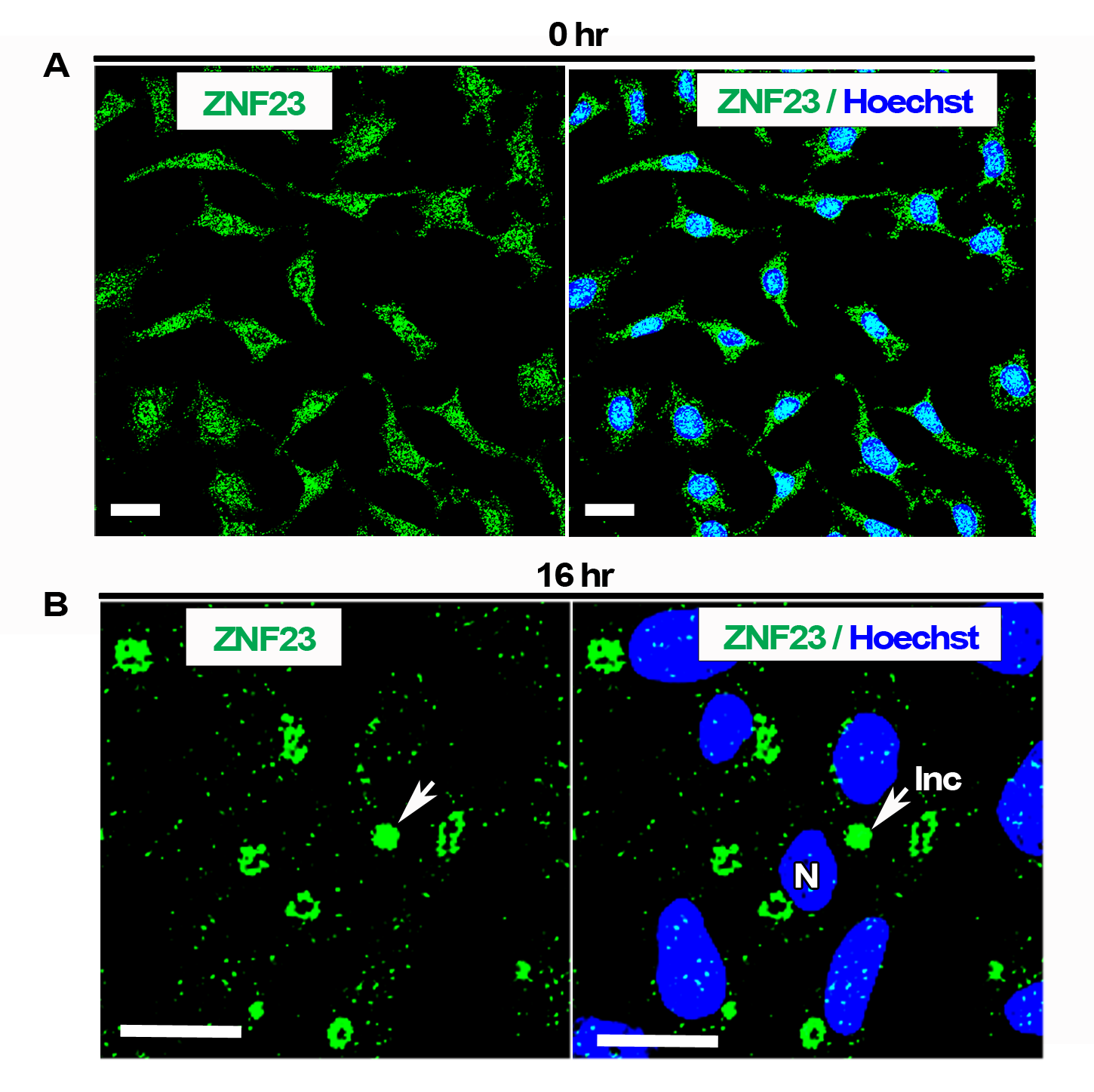

Supplement: Figure S6 — Redistribution of ZNF23 from the nucleus and cytosol into the inclusion lumen during Ct development. HeLa cells were grown on coverslips in cell culture medium without antibiotics and cycloheximide. At near confluence, some cells were fixed (panel A, 0 hr) or were infected with Ct strain E at a MOI of 1 and were fixed 16 hr post-infection (panel B). Human ZNF23 protein was detected with a rabbit antibody stained with an AlexaFluor®488-conjugated anti-rabbit antibody (green). DNA was stained with the Hoechst 33258 dye (blue). Images were taken with a Zeiss LSM710 confocal microscope at a magnification of 40x in panel A and of 63x in panel B. The bars in the panels represent 20 µm. Note that the bacteria were only detected by staining their DNA with the Hoechst dye. The results obtained for staining of ZNF23 and the merged image obtained with stained DNA are shown. On panel B, an inclusion (Inc) and a nucleus are indicated by an arrow and with a capital N, respectively. In un-infected cells (panel A), ZNF23 is located in the cytosol and nucleus. In infected cells, ZNF23 has been recruited into the inclusions and is no longer detected in the host cell. (TIF) [file pone.0036843.s006.tif]
